# The impact of altered gut microbiota and lipid metabolism on the progression of endometrial cancer in overweight populations

**DOI:** 10.3389/fendo.2025.1610534

**Published:** 2025-07-31

**Authors:** Jiayu Chen, Haochen Peng, Yawen Shao, Zhenzhen Wu

**Affiliations:** 1 Gansu Province Maternal and Child Health Hospital (Gansu Central Hospital) Women's Health Department, Lanzhou, China; 2 First School of Clinical Medicine, Gansu University of Chinese Medicine, Lanzhou, China

**Keywords:** overweight, endometrial cancer, gut microbiota, gut microbial ecosystem, lipid metabolism

## Abstract

**Background:**

Endometrial cancer (EC) is one of the common malignant tumors among women, and in recent years, the role of gut microbiota in tumorigenesis has been increasingly gaining attention.Existing research has shown that the gut microbiome, establishes axis connections with multiple extra-intestinal organs. However, whether gut microbes affect the process of endometrial carcinogenesis through metabolic pathways and the specific mechanisms by which they promote the development of EC remain unclear. This study aims to explore the impact of overweight-mediated gut microbiota on the initiation or progression of EC and to assess its relationship with metabolites, thereby providing new insights for early diagnosis and treatment.

**Methods:**

In this study, we analyzed gut microbiota differences among normal-weight, overweight EC patients, and healthy controls using 16S rRNA sequencing. Liquid chromatography-mass spectrometry (LC-MS) and KEGG analysis identified group-specific metabolites and pathways, while Spearman correlation analysis revealed associations between microbiota and metabolites.

**Results:**

This study revealed that in the ECMO group, the genus Megamonas exhibited the highest abundance and significant intergroup differences (H=13.46, P<0.05). Additionally, the *Bacillota/Bacteroidota* ratio (B/B ratio) gradually increased in the CN, ECMN, ECMO group. LEfSe analysis identified *Megamonas* and Amedibacillus as potential biomarkers for the ECMO group. Serum metabolomics of overweight EC patients highlighted lipid metabolism-related metabolites with the most specific expression. KEGG enrichment analysis of differential metabolites highlighted that the Glycerophospholipid metabolism and Purine metabolism pathways were notably significant in both the ECMN and ECMO groups.

**Conclusion:**

The study found significantly elevated abundance of *Megamonas* in the gut microbiota of overweight EC patients, which may promote EC progression by degrading inositol to enhance lipid absorption. This reveals the role of gut microbiota in EC pathogenesis through lipid metabolism regulation, providing a theoretical basis for microbiota-based diagnostic and therapeutic strategies.

## 1 Introduction

Endometrial Cancer (EC) is one of the top five most common malignant tumors in women, with approximately 320,000 new patients diagnosed globally each year (1). The Bokhman classification (2) categorizes EC into two histological subtypes: Type I and Type II. Type I is primarily associated with obesity and other components of metabolic syndrome, which have been identified as independent risk factors for the development of EC. Type II tumors exhibit more aggressive behavior and are not estrogen-driven. However, studies have shown (3) the complexity of EC risk factors, with obesity playing a significant role in both EC subtypes. The dramatic rise in obesity prevalence is driven by sedentary lifestyles, increased caloric intake, and widespread polygenic susceptibility (genetic predisposition involving multiple genes). Recent research (4) suggests that sustained weight loss, which reverses obesity-induced metabolic alterations and gut dysbiosis, can significantly reduce the risk of developing EC.

Microorganisms,by colonizing diverse niches of the human body, establish an intimate symbiotic relationship with their host, collectively forming a holobiont (meta-organism). As an integral component of human biology, the microbiota may directly or indirectly modulate cancer susceptibility and tumor progression (5, 6). The homeostatic state of the gut microbiota has been demonstrated to critically influence systemic health (7). For instance, gut microbiota can influence host physiological functions in multiple ways, such as affecting intestinal wall permeability, defending against pathogenic microbial invasion, and releasing anti-inflammatory or pro-inflammatory factors, thereby increasing the risk of carcinogenesis.Japanese researchers propose that gut microbiota diversity is significantly higher in obese populations compared to non-obese individuals, potentially linked to ethnicity, geographic region, and other factors (8). The development of EC may be linked to abnormal estrogen levels and adverse consequences of overweight, potentially driven by modern lifestyle changes such as improved quality of life and dietary patterns. Additionally, disrupted nutrient absorption due to gut dysbiosis may contribute to obesity, which in turn drives conditions such as hypertension, diabetes, and hormonal imbalances-all recognized risk factors for EC (9). Therefore, this study, based on 16S rRNA high-throughput sequencing and LC-MS non-targeted metabolomics, aims to explore the differential gut microbiota and serum metabolites in overweight individuals with EC, with the goal of providing effective strategies for the prevention and treatment of EC.

## 2 Materials and methods

### 2.1 Inclusion and exclusion criteria, and grouping

This study was conducted at the Gansu Provincial Maternity and Child Health Care Hospital from February 2023 to October 2023. Through rigorous screening based on inclusion and exclusion criteria, a total of 17 EC patients and 22 healthy controls(all of normal weigh, CN group) were enrolled in the study. The EC cohort included an EC normal-weight group (ECMN group, n=8) and an EC overweight/obese group (ECMO group, n=9). We used body mass index (BMI) for grouping. According to the World Health Organization (WHO) criteria, overweight is defined as a BMI greater than or equal to 25 kg/m^2^ and a normal weight is defined as a BMI less than 25 kg/m^2^.

The inclusion criteria were as follows: Experimental Group: (1). Individuals aged between 20 and 60 years. (2). Histopathological type confirmed as endometrioid adenocarcinoma. No history of diarrhea or other gastrointestinal diseases within 4 weeks prior to enrollment. All participants included in the study had signed informed consent forms and agreed to comply with the study protocol. Exclusion Criteria: (1) A history of estrogen-dependent diseases. (2) A history of other malignant tumors. (3) Pregnant or breastfeeding women. (4) Patients who have received chemotherapy, radiotherapy, or cellular immunotherapy before surgery. (5) Use of antibiotics, probiotics, or other medications affecting gastrointestinal function within 3 months before enrollment. (6) Use of immunosuppressants or hormonal 3treatments within 3 months before enrollment. (7) A history of diarrhea or other gastrointestinal diseases within 3 months before enrollment. (8) Vegetarians or individuals with special dietary habits. (9) Exclude participants with a history of postmenopausal hormone replacement therapy or use of hormonal contraceptives.

### 2.2 Sample collection

Before surgery or on the day of the health checkup for the healthy control group, participants collected stool samples approximately the size of a soybean (about 1.5 to 3 grams), which were placed in 7 mL sterile collection tubes and immediately stored at -80°C in a deep freezer. Each participant also provided 2.5 mL of venous blood, which was promptly sent to the laboratory. The blood was centrifuged at 4000 rpm for 5 minutes at room temperature to separate the serum. The separated 1 mL serum was aliquoted into two cryovials, labeled, and quickly stored at -80°C for subsequent serum metabolite analysis.

### 2.3 Library preparation and sequencing

Genomic DNA was extracted from fecal samples using the MagPure Stool DNA KF Kit B. The PCR amplification was carried out using specific primers 338F (ACTCCTACGGGAGGCAGCAG) and 806R (GGACTACHVGGGTATCTAAT). The reaction mixture had a total volume of 20 μL, containing 30 ng of quality-verified genomic DNA and fusion primers. The V3–V4 region of the 16S rRNA gene was PCR-amplified: initial denaturation at 98°C for 1 minute, followed by 30 cycles consisting of denaturation at 98°C for 10 seconds, annealing at 50°C for 30 seconds, and extension at 72°C for 60 seconds; a final extension step was performed at 72°C for 10 minutes. Amplicons were purified with Agencourt AMPure XP beads to remove primer dimers, followed by library construction using the DNA Fragment Size Selection Bead-Based Kit (LB00V60) for adapter ligation and size selection of target fragments (400–500 bp). Purified products were dissolved in Elution Buffer, and library fragment distribution/concentration (>4 nM) was verified using an Agilent 2100 Bioanalyzer. Qualified libraries underwent paired-end sequencing (2×250 bp) on an Illumina MiSeq platform, with filtered data used for downstream bioinformatic analysis.

### 2.4 Data filtering and bioinformatics analysis

#### 2.4.1 Data filtering and tag linking

The raw sequencing data were initially filtered. A sliding window strategy with a 25-bp window was applied. If the average quality score within the window was below 20, both the window and its subsequent sequences were discarded. If the trimmed read length was <75% of the original, the read was discarded. Adapter contamination was removed by setting the overlap region between adapters and reads to 15 bp, allowing for 3 mismatches. Reads containing N bases and low-complexity reads (defined as sequences with ≥10 consecutive identical bases) were also removed. Finally, samples were identified based on barcode and primer sequences, with no mismatches allowed between the barcode and sequencing reads.

Sequence assembly was conducted with FLASH (Fast Length Adjustment of Short Reads, v1.2.11), which utilized overlapping relationships to assemble paired-end reads into a single sequence, yielding high-variable region tags. The assembly conditions included: (1) a minimum matching length of 15 bp; (2) an overlap region mismatch rate of 0.1 (for detailed data, see the appendix).

#### 2.4.2 OTU clustering analysis

USEARCH software (v7.0.1090) was employed to cluster the assembled tags into operational taxonomic units (OTUs). Initially, UPARSE was used to perform clustering at 97% similarity, resulting in the representative sequences for each OTU. Subsequently, UCHIME (v4.2.40) was employed to detect and remove chimeras that may have been generated during the PCR amplification process. For 16S and ITS sequences, chimera removal was achieved by aligning the sequences with established chimera databases. The 16S chimera database used was the gold database (v20110519), while the ITS chimera database used was UNITE (v20140703). Depending on the sequencing region, comparisons were performed against the full-length ITS, ITS1, or ITS2 regions. For 18S sequences, a *De novo* method was used to remove chimeras. Lastly, the usearch_global method was applied to align all tags with the OTU representative sequences, generating an OTU abundance table for each sample.

#### 2.4.3 Microbial community diversity analysis

Microbial community diversity in the gut was analyzed using QIIME2 (v2023.2) to compute the Shannon index, Chao index, and observed OTUs, which were used to assess alpha diversity. Beta diversity analysis was conducted using the Bray-Curtis distance matrix and weighted UniFrac distance. Principal coordinates analysis (PCoA) was utilized to visualize the differences in community structure among groups. All analyses were performed in R (v4.4.1) with the vegan package (v2.6-4), and a significance threshold was set at P < 0.05.

#### 2.4.4 Microbial community differences and linear discriminant analysis

DESeq2 (v1.12.4) was employed to analyze the microbiota’s relative abundance data at the phylum, family, and genus levels. A negative binomial distribution model was used to detect differentially abundant taxa between groups, using the criteria of FDR-corrected P < 0.05 and |log2FoldChange| > 2. Additionally, LEfSe analysis (LDA score > 3.0) was conducted to identify microbial biomarkers that significantly contribute to the differences between groups.

### 2.5 Metabolomics analysis

#### 2.5.1 Serum sample extraction

Metabolites in serum samples were analyzed using a high-resolution mass spectrometer in both positive and negative ion modes. Peaks were detected using XCMS software, and preliminary identification was performed based on the mass-to-charge ratio (m/z) 44and retention time. Subsequently, metaX software was employed to match the detected metabolites against the HMDB and KEGG databases, providing primary identification results. To improve accuracy, a secondary mass spectrometry library was used to compare the sample data, yielding high-confidence metabolite identification results. For data processing, quality control was applied to remove low-quality peaks (with QC sample missing more than 50% or actual samples missing more than 80%). Missing values were imputed using the K-Nearest Neighbors (KNN) method, and normalization was performed using the Probabilistic Quotient Normalization (PQN) method. Finally, metabolite identification and relative quantification results were obtained.

#### 2.5.2 Serum metabolite detection

The samples were sequentially arranged in the ultra-high pressure liquid chromatography system, and pre-separation was carried out using an ACQUITY UPLC BEH T3 column. The chromatographic conditions were set as follows: column temperature at 50°C, flow rate at 0.3 mL/min, with mobile phase A being a 0.1% formic acid aqueous solution, and mobile phase B being a 0.1% formic acid-acetonitrile solution. The gradient elution program is detailed in the appendix.

Mass spectrometry data were collected using a Q-Exactive high-resolution mass spectrometer, operating alternately in positive and negative ion modes. The ion source parameters were as follows: sheath gas pressure at 10 psi, auxiliary gas pressure at 40 psi, ion transfer tube temperature at 350°C, and spray voltage at +3800 V (positive ion mode)/-3100 V (negative ion mode). Data-dependent acquisition (DDA) mode was applied, with a full scan range of 70–1050 Da (resolution 70,000 @ m/z 200), automatically selecting the top 3 precursor ions with signal intensity >1 × 10^5^ for fragmentation (resolution 17,500 @ m/z 200). QC samples were introduced after every 10 samples to monitor system stability, and systematic errors were corrected by normalizing the inter-sample variation using the QC samples.

#### 2.5.3 Metabolomics data processing and analysis

The data processing workflow is as follows: First, metabolites with missing values exceeding 50% in QC samples or 80% in actual samples are excluded during quality control. Subsequently, missing values are imputed using the K-Nearest Neighbors (KNN) method. Data normalization is performed using Probabilistic Quotient Normalization (PQN), referencing the median peak area ratio from the QC samples. The identified metabolites were annotated using the KEGG database, and significant differential metabolites were selected using a PLS-DA model. The selection criteria were: fold change > 1.5 or < 1/1.5, P < 0.05, and VIP > 1. Finally, KEGG pathway enrichment analysis was conducted on the differential metabolites, with P < 0.05 considered significant.

### 2.6 Statistical analysis

Clinical data were analyzed using SPSS 26.0 software. Normally distributed continuous variables were expressed as Mean ± SD, and between-group comparisons were performed using Tukey-Kramer one-way ANOVA. Non-normally distributed data were expressed as median (interquartile range), and comparisons between groups were performed using the Kruskal-Wallis rank sum test. Categorical data were analyzed using the Chi-square test. Spearman correlation analysis was used to assess the correlation between specific bacterial taxa and specific metabolites. A P value < 0.05 was considered statistically significant.

## 3 Results

### 3.1 Baseline data analysis

This study selected 17 patients diagnosed with endometrioid adenocarcinoma (EC group) based on postoperative pathological diagnosis, and 22 healthy women undergoing physical examination at the same time as the control group, according to the inclusion and exclusion criteria. The EC group was further divided into overweight patients with endometrioid adenocarcinoma (ECMO group) and normal-weight patients with endometrioid adenocarcinoma (ECMN group). Clinical characteristics such as age, weight, BMI, waist circumference, blood pressure, and menopause status were compared among the three groups (**Table 1**).

**Table 1:** Baseline characteristics of study groups.

Variable	ECMO group (n=9)	ECMN group (n=8)	Control group (n=22)	P-value
Age (years)	48.78 ± 9.02	52.5 ± 4.38	33.87 ± 7.28	0.29
Height (m)	1.6 ± 0.05	1.59 ± 0.03	1.61 ± 0.04	0.99
Weight (kg)	83.11 ± 12.4	57.88 ± 5.91	55.13 ± 6.53	<0.001*
BMI (kg/m²)	32.24 ± 3.37	22.79 ± 1.89	21.31 ± 2.81	0*
Waist circumference (cm)	96.28 ± 21.12	94.25 ± 29.09	74.27 ± 6.51	0.23
Hip circumference (cm)	101.06 ± 23.14	90.44 ± 10.82	92.72 ± 5.87	0.56
Diabetes mellitus (n, %)	3	1	0	0.07
Hypertension (n, %)	5	3	0	0.02*
After menopause (n, %)	5	5	1	0.11
CA125 (u/ml)	35.09 ± 48.31	25.45 ± 12.81	–	0.98
CA199 (u/ml)	27.51 ± 31.11	24.46 ± 21.0	–	0.99
CA153 (u/ml)	14.66 ± 9.87	10.09 ± 4.38	–	0.28
HE4 (pmol/L)	98.61 ± 59.14	79.16 ± 43.06	–	0.09

EC, Endometrial cancer.

*Statistically significant (P < 0.05).

In terms of BMI, the ECMO group had significantly higher BMI than both the ECMN group and the control group (P < 0.05), with 9 patients (52.9%) in the ECMO group. Regarding hypertension, the number of patients in the ECMO group was significantly higher than in the other two groups (P < 0.05). There were no significant differences between the two groups regarding menopause, but 10 patients (58.8%) in the EC group were in a postmenopausal state. There were no significant differences in the tumor markers CA125 and HE4 between the ECMO group and the ECMN group (P > 0.05).

### 3.2 Gut microbiota diversity analysis across different groups

**Figure 1:**
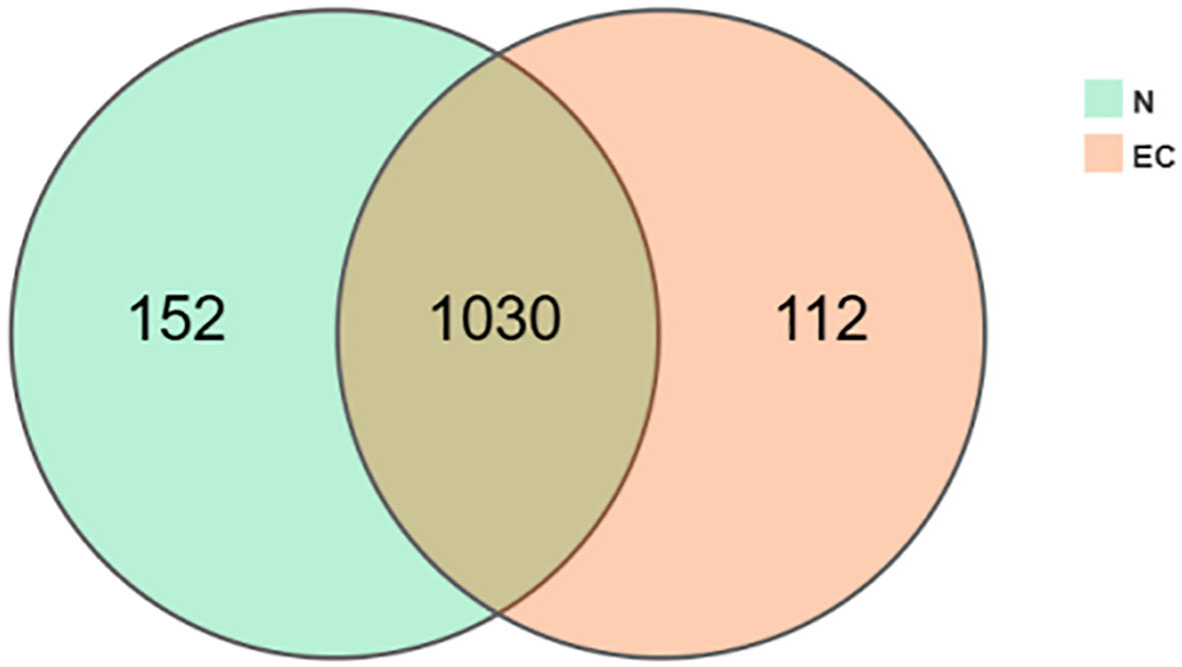
The distribution of OTUs between the two groups.

As shown in **Figure 1**, the Venn diagram displays a total of 1294 OTUs, with 152 unique OTUsin the CN group and 112 unique OTUsin the EC group. To thoroughly investigate the gut microbiota characteristic profiles of overweight/obese populations with EC, we conducted pairwise comparative analyses between three groups: CN vs. ECMN and ECMN vs. ECMO.

**Figure 2:**
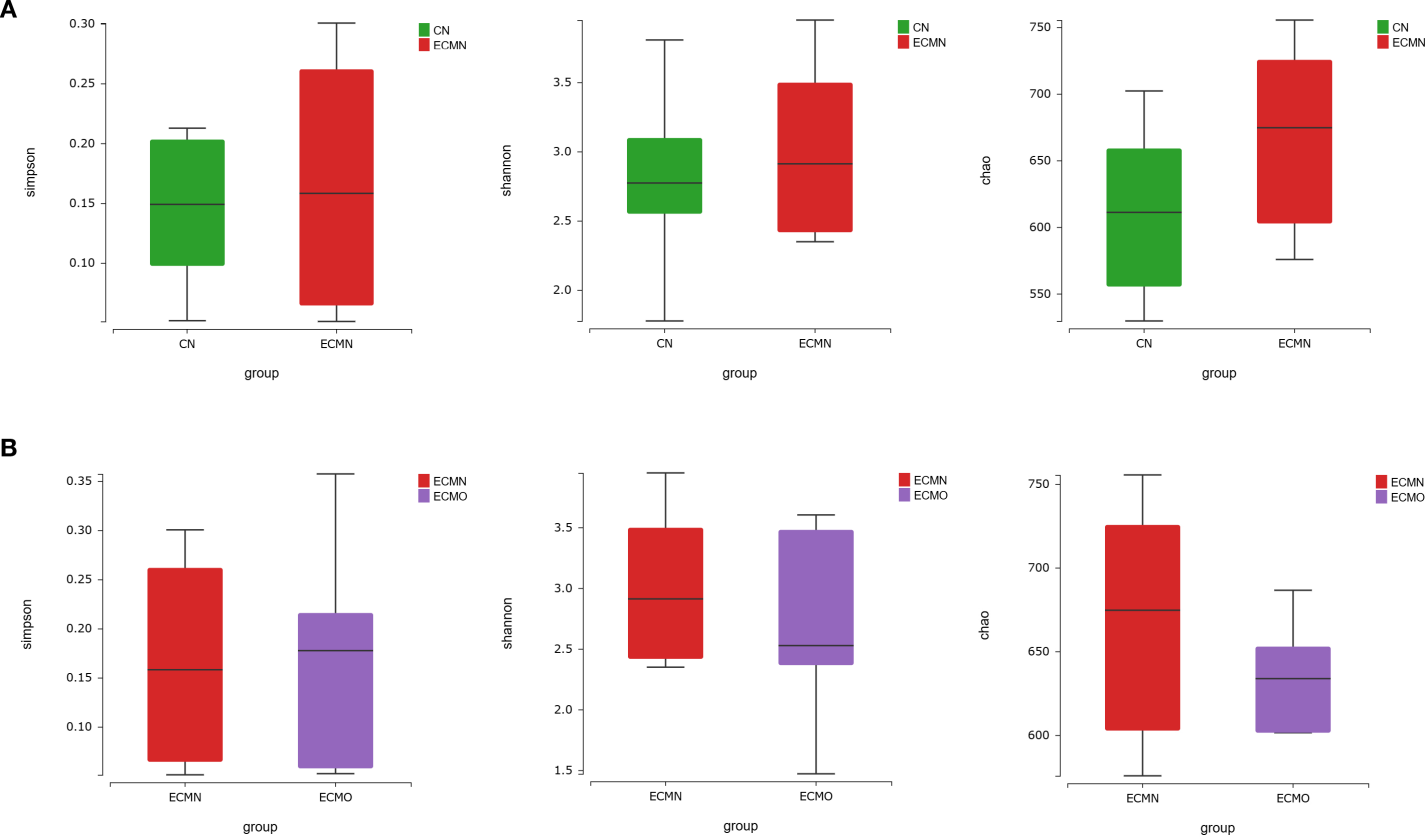
The comparison of alpha diversity between the three groups. **(A)** (CN vs ECMN) and **(B)** (ECMN vs ECMO).

Alpha diversity analysis revealed no statistically significant differences between the CN and ECMN groupsin Chao1 (P = 0.23), Shannon (P = 0.67), or Simpson indices (P = 0.96) (**Figure 2A**). For the ECMN vs. ECMO comparison, the Shannon (P = 0.56) and Simpson indices (P = 0.94) showed no significance, while the Chao1 index was statistically significant (P = 0.03) (**Figure 2B**).

**Figure 3:**
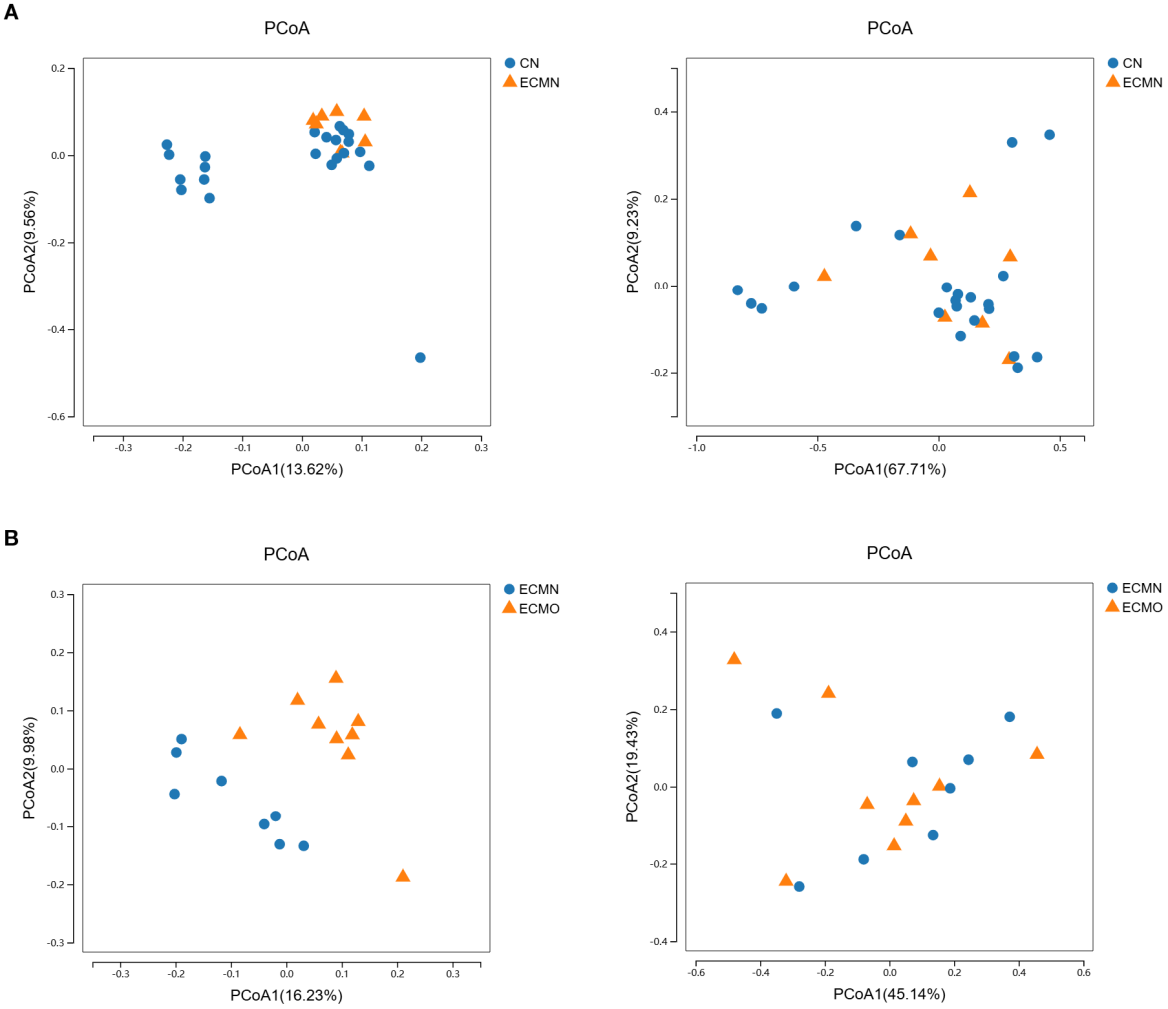
The clustering of gut microbial communities among the three groups. **(A)** (CN vs ECMN) and **(B)** (ECMN vs ECMO).

To further visualize gut microbiota differences among all study groups, unweighted PCoA analysisrevealed significant separation between the CN and ECMN groups (P < 0.0001) and between the ECMN and ECMO groups (P < 0.0001) (**Figure 3A**). In contrast, weighted PCoA analysisshowed no significant separation between any groups (P > 0.05) (**Figure 3B**). Unweighted analyses primarily account for the presence or absence of species, whereas weighted analyses incorporate relative abundance of species. This discrepancy may indicate that, despite structural differences in community composition (captured by unweighted methods), there are similarities in species abundance distributions across groups.

### 3.3 Gut microbiota composition analysis

**Figure 4:**
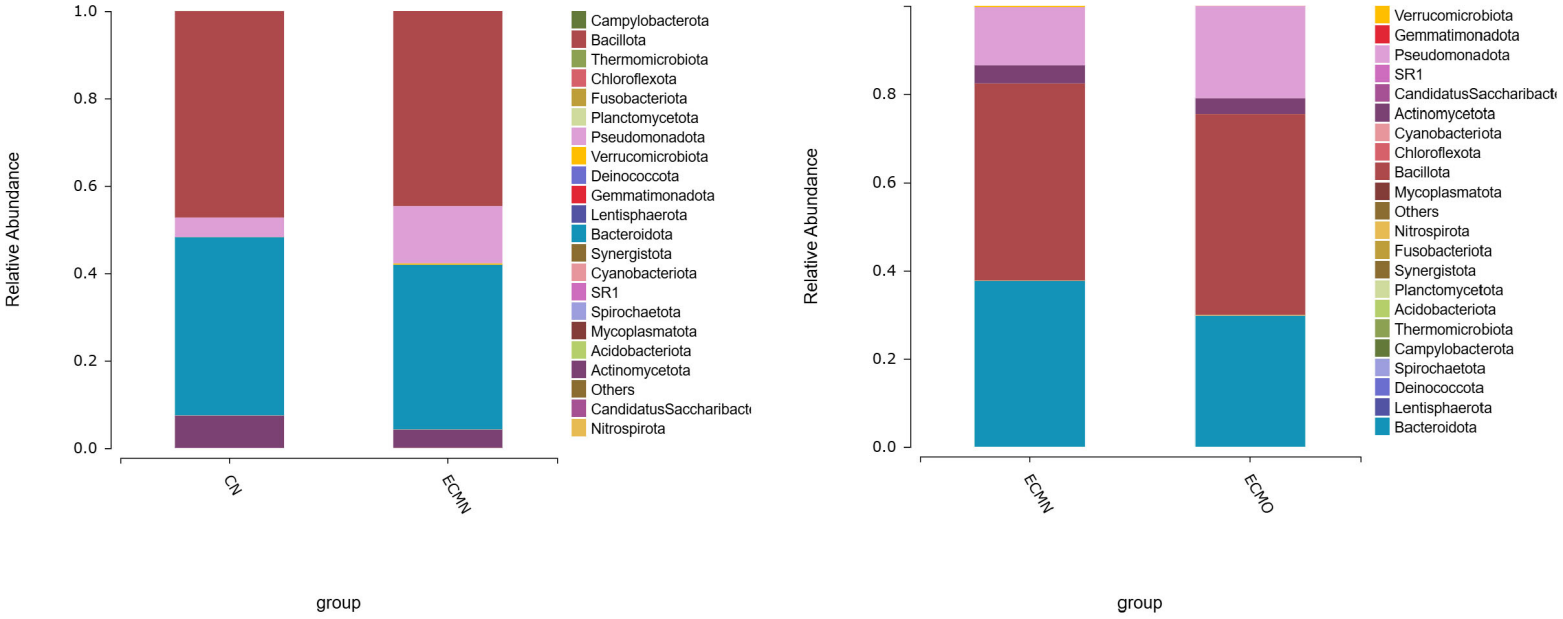
The relative abundance (%) of gut microbiota at the phylum level across the three groups.

At the phylum level (**Figure 4**), *Bacillota* was the most abundant phylum in the CN (47.91%), ECMN (44.43%), and ECMO (47.25%) groups. In contrast, the relative abundances of *Bacteroidota* and *Actinomycetota* gradually decreased from the CN to ECMN and ECMO groups, whereas *Pseudomonadota* progressively increased in the CN (4.50%), ECMN (13.13%), and ECMO (18.69%) groups.In EC patients, the *Pseudomonadota* abundance was significantly higher in the ECMO group compared to the ECMN group. Furthermore, the *Bacillota/Bacteroidota* (B/B)ratio, a critical marker of microbial dysbiosis, increased from 1.32 in the ECMN group to 1.56 in the ECMO group. Elevated B/B ratios have been linked to dysregulation of inflammatory responses and metabolic pathways (e.g., tryptophan and lipid metabolism), potentiallycontributing to EC progression (10, 11). In the ECMN group, four bacterial phylashowed statistically significant differences. The dominant phylumwas *Pseudomonadota* (H = 8.63, P < 0.05), while *Mycoplasmatota*, *Campylo bacterota*, and *Candidatus Sacchari bacteria* also exhibited significant differences in abundance. In contrast, only *Mycoplasmatota* demonstrated a statistically significant difference in the ECMO group.

**Figure 5:**
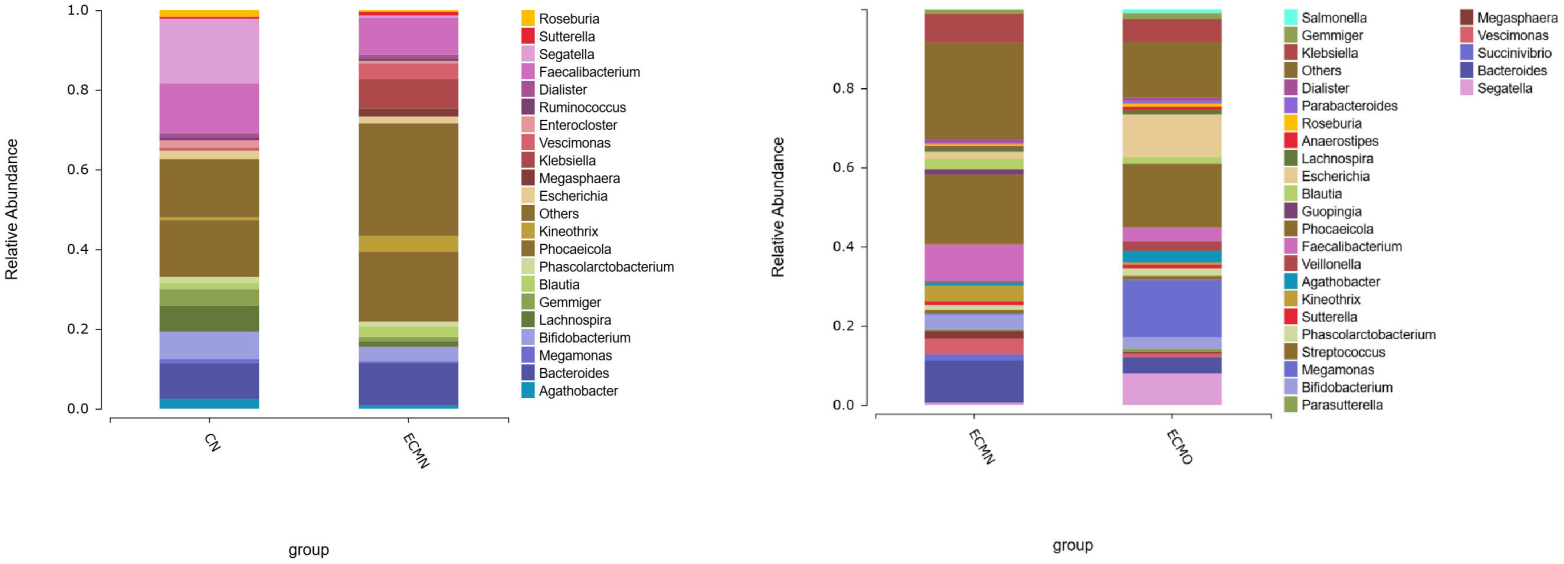
The relative abundance (%) of gut microbiota at the genus level across the three groups.

At the genus level (**Figure 5**), *Phocaeicola* exhibited the highest abundance in the CN group (14.87%), ECMN group (18.12%), and ECMO group (16.49%), but no significant differences were observed in its abundance among the groups. Comparative analysis at the genus level identified 48 bacterial genera with statistically significant differencesamong the three groups. The dominant genera in the ECMO group included*Megamonas* (H=13.46, P<0.05), *Clostridium sensu stricto* (H=0.32, P=0.01), and *Acinetobacter* (H=0.1, P<0.01). In the ECMN group, the dominant genera included *Megasphaera* (H=1.84, P=0.001), *Streptococcus* (H=0.54, P<0.005), *Kineothrix* (H=3.10, P<0.05), and *Vescimonas* (H=3.40, P<0.05). Among these differential genera, *Megasphaera* showed higher abundance in the ECMN group, with significant differences among the three groups. In the ECMO group, *Megamonas* exhibited the highest abundance, with significant differences among the three groups (H=13.46, P<0.05).

### 3.4 LEfSe analysis of gut microbiota

**Figure 6:**
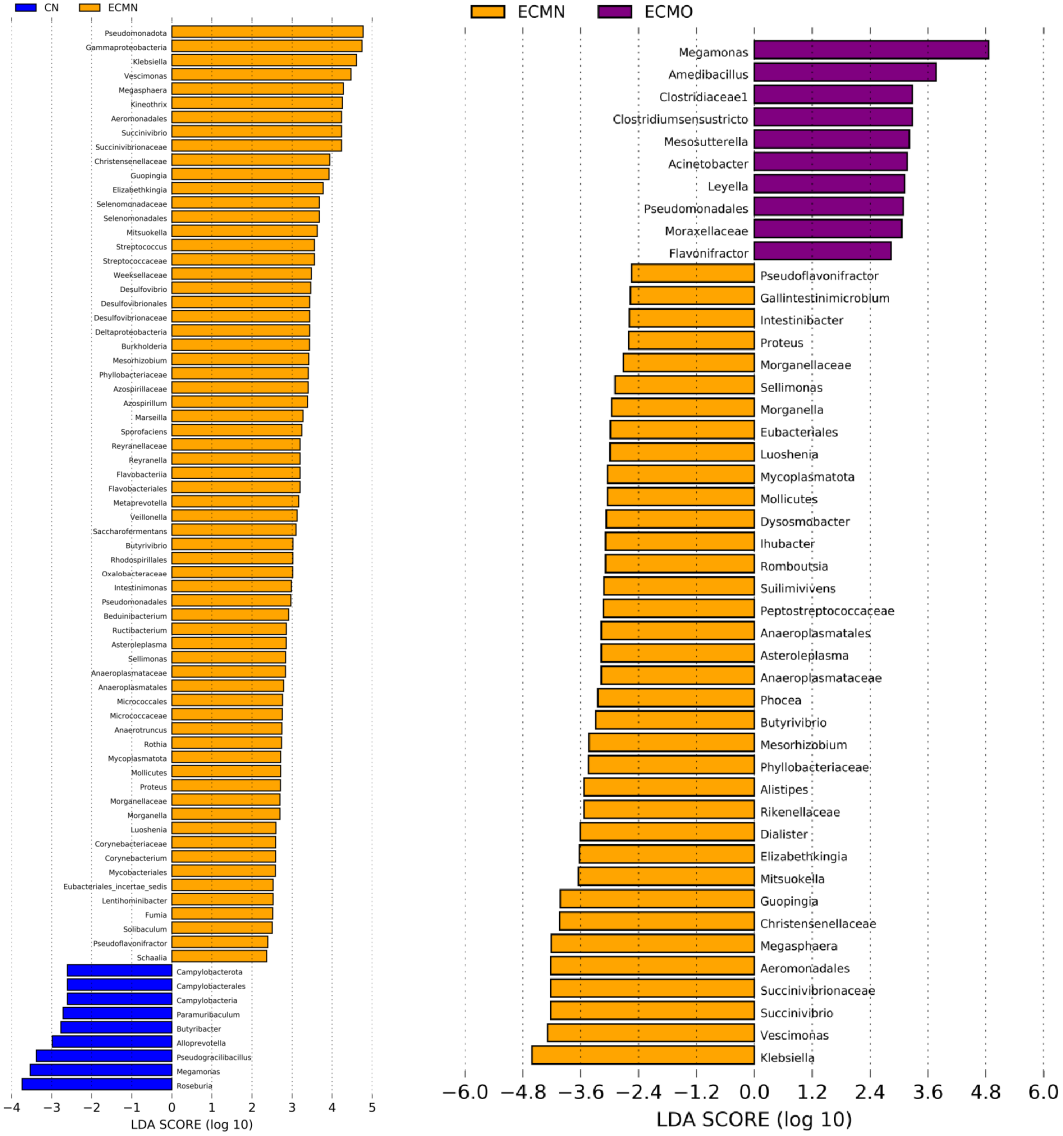
The bacterial taxa with significant differences in abundance across groups.

LEfSe (Linear Discriminant Analysis Effect Size) identifies biologically relevant biomarkers by integrating statistical significanceand biological consistencyacross groups. Building on the LEfSe analysis and taxonomic visualization results, we further validated the differentially abundant taxa in the gut microbiota composition between the ECMN and ECMO groups.Given the high discriminative power at the genus level, the identified genera were selected as candidate biomarkers for further investigation. The results revealed distinct:the ECMO group, the genera with the greatest impact on the gut microbiota structure,ranked by linear discriminant analysis(LDA) scores, were: *Megamonas*, *Amedibacillus*, *Clostridium sensu stricto*, *Mesosutterella*, *Acinetobacter*, *Leyella*, and *Flavonifractor*. In the ECMN group, the genera with higher LDA scores included: *Klebsiella*, *Vescimonas*, *Megasphaera*, *Kineothrix*, *Succinivibrio*, *Guopingia*, and *Elizabethkingia*(**Figure 6**). These results confirm the enrichment of the above-discussed characteristic generain their respective groups, solidifying *Megamonas* and *Amedibacillus* as potential biomarkersfor the ECMO group, and *Klebsiella* and *Vescimonas* for the ECMN group.

### 3.5 Analysis of differential metabolites and metabolic pathway enrichment in overweight EC patients

**Figure 7:**
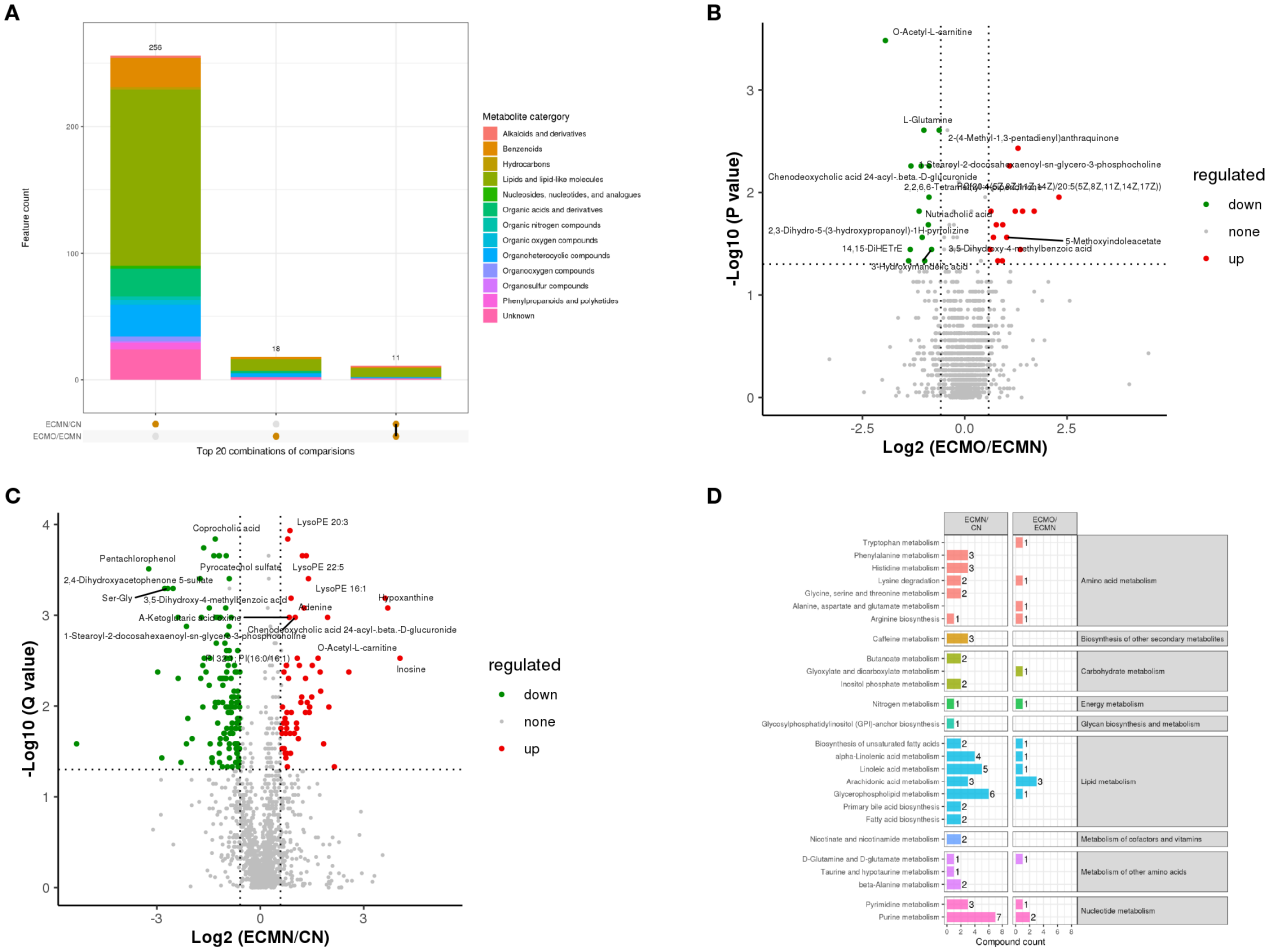
**(A)** illustrates the quantity and classification of secondary differential metabolites detected in the three groups, **(B, C)** depict the divergent metabolic profilesbetween groups, **(D)** presents the KEGG pathway enrichment analysis, confirming the central role of lipid metabolism.

To investigate the serum metabolic characteristics of overweight EC patients, we analyzed differential metabolites and associated metabolic pathways between the ECMN and ECMO groups. The results demonstrated significant differences in metabolic profilesbetween both EC groups and the CN group, highlighting obesity-driven metabolic dysregulation in EC pathogenesis.Using the criteria of VIP > 1, P < 0.05, FC > 2, or FC < 0.5, hierarchical clustering was performed based on the expression levels of significantly differential metabolites. The analysis identified 4198 differential ions in the ECMN group (1352 upregulated and 2796 downregulated metabolites) and 406 differential ions in the ECMO group (216 upregulated and 190 downregulated metabolites). All differential metabolites were validated through rigorous quantitative quality control (QC) procedures. Statistical results for secondary differential metabolites are shown in **Figure 7**.

This study found that the secondary differential metabolites of EC primarily originate from lipids and lipid-like molecules. Further screening of highly expressed metabolites in the ECMN and ECMO groups, followed by ranking based on Variable Importance in Projection (VIP) scores, demonstrated the following key findings: In the ECMO group, O-Acetyl-L-carnitine, Chenodeoxycholic acid 24-acyl-β-D-glucuronide, and 3-Hydroxymandelic acid were the most significantly expressed metabolites. In the ECMN group, Pentachlorophenol, 2,4-Dihydroxyacetophenone 5-sulfate, and Benzoylcholine were the most significantly expressed metabolites. KEGG enrichment analysis of the differential metabolites indicated that the Glycerophospholipid metabolism and Purine metabolism pathways were notably significant in both the ECMN and ECMO groups. Additionally, the primary metabolic pathways of EC were predominantly concentrated in Lipid metabolism.

### 3.6 Correlation between gut microbiota and metabolites in overweight EC patients

**Figure 8:**
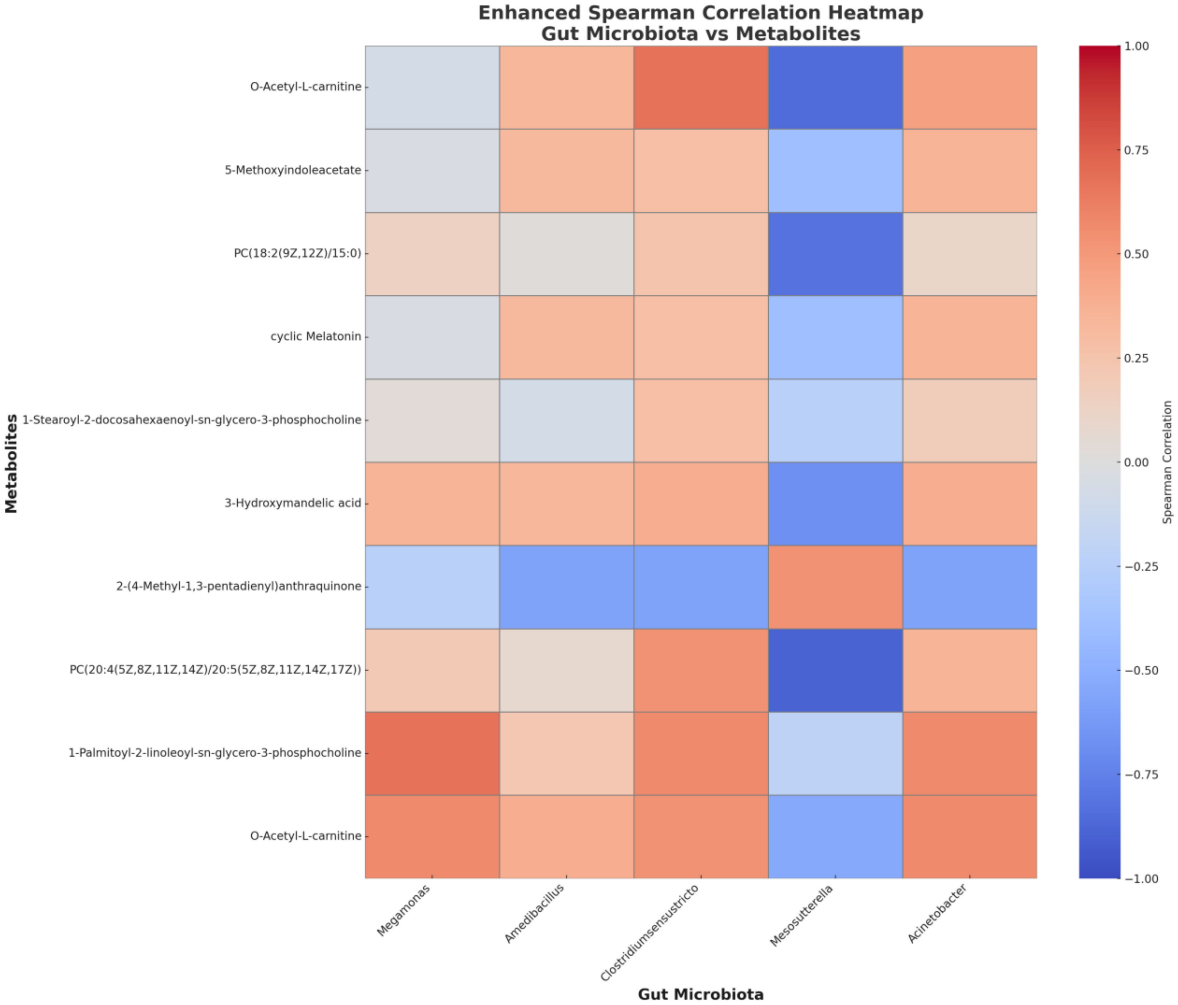
The Spearman correlations between bacterial genera and metabolites in the ECMO group.

This study analyzed the correlation between five specific gut microbial genera in the ECMO group and the top 10 specific metabolites ranked by VIP values. The results showed the following in the Spearman correlation analysis between gut microbial genera and metabolites: *Megamonas* exhibited significant positive correlations with O-Acetyl-L-carnitine and 1-Stearoyl-2-docosahexaenoyl-sn-glycero-3-phosphocholin (r = 0.57, 0.67, respectively). *Clostridium sensu stricto* showed a significant positive correlation with O-Acetyl-L-carnitine (r = 0.679). *Mesosutterella* demonstrated significant negative correlations with multiple metabolites, including PC(20:4(5Z,8Z,11Z,14Z)/20:5(5Z,8Z,11Z,14Z,17Z)), Acetyl-L-carnitine, PC(18:2(9Z,12Z)/15:0), and 3-Hydroxymandelic acid(r = -0.89, -0.85, -0.82, -0.67, respectively)(**Figure 8**). The analysis of metabolite categories revealed that lipids and lipid-like molecules constituted the predominant class of differential metabolites, with organic acids and derivatives also playing a significant role in the correlation network. These findings highlight the potential mechanisms by which specific gut bacterial genera regulate key metabolites and underscore the intricate interplay between gut microbiota and host metabolism in health and disease.

## 4 Discussion

With the advancement of gut microbiota research, the mechanisms linking it to tumorigenesis have become increasingly clear. Current studies indicate that gut dysbiosis may contribute to carcinogenesis through multiple pathways, including disruption of intestinal mucosal barrier integrity and induction of metabolic disturbances(e.g., estrogen imbalance and reduced short-chain fatty acids [SCFAs]) (12). Specifically, the core mechanisms by which microbial dysbiosis drives tumor initiation and progression include:(1) activation of chronic inflammatory signaling via microbial-associated molecular patterns (MAMPs) binding to pattern recognition receptors (e.g., TLRs); (2) direct damage to host genomic stability or epigenetic regulation by microbial metabolites (e.g., secondary bile acids and polyamines); and (3) immune tolerance triggered by molecular mimicry between microbial antigens and tumor antigens (13–16). Notably, microbiota-mediated carcinogenic mechanisms exhibit significant heterogeneity across different tumor types, reflected both in dynamic shifts in specific bacterial taxa (e.g., pathogenic overgrowth or depletion of commensals) and tissue-specific activation of microbiota-host interaction pathways. For example, in colorectal cancer, specific pathogens such as colibactin-producing bacteria directly induce DNA double-strand breaks, while adhesin-receptor interactions activate pro-proliferative signaling pathways (e.g., Wnt/β-catenin), synergistically driving epithelial cell malignant transformation (17). Metagenomic studies have further revealed that tumor progression is frequently accompanied by a reduction in gut microbiota alpha diversity and the enrichment of signature bacterial genera. For instance, the aberrant proliferation of taxa such as *Bacteroides*and *Parvimonas*—observed in colorectal cancer—suggests that these microbes may serve as biomarkers implicated in the multistage transition from precancerous lesions to invasive malignancies (18).

Recent research has confirmed that dysbiosis of the gut microbiota is closely linked to the onset and progression of EC, with its mechanisms interacting synergistically with metabolic abnormalities such as obesity, hypertension, and diabetes. Studies have highlighted the critical role of obesity in promoting EC. Excess adipose tissue, particularly in obese individuals, increases aromatase activity, which converts androgens into estrogens, thereby raising estrogen levels (19). One study (20) underscores how metabolic disruptions, including insulin resistance, play a significant role in EC progression, reinforcing the notion that obesity is a major risk factor for EC. Obesity-induced visceral fat accumulation also leads to the secretion of pro-inflammatory cytokines, such as IL-6, which activate chronic systemic inflammation (21). This inflammatory response not only contributes to the increased risk of EC but also promotes immune evasion and cellular proliferation through various signaling pathways. Research (22) has shown that this inflammation affects the tumor microenvironment, thereby facilitating cancer cell growth and enhancing tumor progression.

Moreover, obesity causes disruptions in lipid metabolism. Excessive fat accumulation leads to energy surplus and fat deposition, which can directly promote the growth and survival of EC cells (23). Alterations in insulin and adipokine signaling, in particular, are crucial pathways through which obesity accelerates EC progression (24). These findings suggest that targeting lipid metabolism could be a promising therapeutic approach for managing EC, particularly in obese patients.Clinical studies have further elucidated the strong association between obesity and increased EC risk. One such study revealed that women with a BMI > 35 kg/m² have a 4.7-fold higher risk of developing EC compared to women with a normal weight (OR=4.7, 95%CI 3.2-6.9). In these individuals, the gut microbiota is notably altered, with a significant increase in the Firmicutes to Bacteroidetes (B/B) ratio. This suggests that the gut microbiota plays a crucial role in the development of obesity-related EC (25). These findings support the hypothesis that the gut microbiota-metabolism axis may act as a key regulatory network for obesity-related EC, where changes in microbial composition influence systemic metabolism and drive the progression of endometrial cancer (26, 27).

Through integrated 16S rRNA high-throughput sequencing and metabolomic profiling, this study reveals a significant association between gut microbiota dysbiosis and lipid metabolic disturbances in overweight/obese populations with EC. In overweight EC patients (ECMO Group), the abundance of the *Megamonas* genus was abnormally elevated, andthe B/B ratio increased progressively with obesity severity—a pattern highly consistent with obesity-associated microbial dysbiosis (10, 28). These findings suggest that gut microbiota dysregulation may serve as a critical biological link between obesity and EC development (12). In particular, the abundance of *Megamonas* exhibits a dose-dependent positive correlation with obesity severity (29, 30). Furthermore, a large-scale metagenomic study of Chinese obese individuals (n=1,005) by Wu et al. (31) confirmed that *Megamonas* enrichment is independently associated with obesity risk (OR=1.78, 95% CI 1.32–2.41). Our study identified its specific enrichment in the EC overweight/obese cohort, and the covariation between *Megamonas* abundance, lipid metabolism dysregulation, and estrogen level abnormalities further supports the potential role of this genus in obesity-associated EC.

The gut microbiota plays a critical regulatory role in the pathogenesis of EC through the interaction of its metabolites with host lipid homeostasis. SCFAs, such as acetate and propionate, inhibit lipolysis by activating the GPR43 receptor. However, excessively high concentrations of SCFAs may paradoxically exacerbate obesity by promoting adipocyte proliferation—via PPARγ-mediated lipid accumulation and AMPK signaling suppression (32, 33). Metabolomic analysis in this study revealed significant activation of lipid metabolism pathways (e.g., glycerophospholipid metabolism) in EC overweight/obese patients, with strong correlations between gut microbial taxa and host lipid metabolites. Clinical data further demonstrated that women with a BMI >35 kg/m² had a markedly elevated EC risk (OR=4.7) (27), and their gut microbiota exhibited an abnormal B/B ratio, suggesting that microbiota-metabolism axis dysregulation may foster a pro-carcinogenic microenvironment via enhanced lipid absorption, estrogen recirculation, and chronic inflammation (34). For instance, *Megamonas* may promote intestinal lipid absorption through the myo-inositol degradation pathway (PWY-7237) (29, 30), while depletion of *Bacteroidota* likely exacerbates intestinal barrier dysfunction, collectively driving EC progression.

Through LEfSe analysis, this study identified characteristic bacterial genera in EC overweight/obese patients (*Megamonas*, *Amedibacillus*) and normal-weight EC patients (*Klebsiella*, *Vescimonas*). The *Megamonas* genus promotes intestinal lipid absorption and visceral fat accumulation by upregulating iolG enzyme expression via the myo-inositol degradation pathway (PWY-7237), thereby inhibiting the function of host fatty acid transport protein 4 (FATP4). Furthermore, as a β-glucuronidase-positive bacterium, *Megamonas* may enhance estrogen enterohepatic recirculation, synergizing with adipose tissue-derived aromatase activity to induce hyperestrogenemia. It is worth noting that,the enrichment of *Megamonas* showed significant correlations with serum lipid molecules (e.g., palmitoleic acid, eicosadienoic acid), suggesting its direct involvement in host lipid homeostasis regulation through metabolic products (25). Additionally, the positive correlation between elevated B/B ratio and serum triglyceride levels (r=0.62, P<0.001) further supports the synergistic effect of microbiota imbalance and lipid metabolism dysregulation as a hallmark feature in EC overweight/obese populations (35), providing novel directions for microbiota-based early diagnostic strategies.

Zhao et al. (36) further demonstrated that *Ruminococcus* N15 and MGS-17—members of the *Firmicutes* phylum—are significantly enriched in the gut microbiota of EC patients. Their abundance showed strong correlations with elevated serum levels of unsaturated fatty acids, including palmitoleic acid (C16:1) and arachidonic acid (C20:2). This finding posits that specific bacterial taxa may directly contribute to EC progression through lipid metabolic reprogramming. The synergistic interaction between *Ruminococcus* and serum metabolites (triglycerides, TG; low-density lipoprotein, LDL) demonstrates significant iagnosticvalue (37). Mechanistically, *Ruminococcus* activates pro-carcinogenic signaling pathways by modulating host unsaturated fatty acid metabolism (e.g., eicosapentaenoic acid, EPA). Clinically, its abundance is positively correlated with serum TG levels (Pearson’s r = 0.58, P < 0.001) and negatively correlated with high-density lipoprotein (HDL), suggesting its dual role in lipid dysregulation and oncogenic microenvironment formation. Furthermore, the local accumulation of fatty acids such as C16:1 (palmitoleic acid) and C18:1 (oleic acid) in EC tumor tissues, coupled with high TG-driven elevation of estrogen levels, further corroborates the mechanistic link by which gut dysbiosis exacerbates EC risk through a “lipid metabolism-estrogen” cascade.

This study reveals the critical role of gut microbiota dysbiosis in the development of overweight/obesity-associated EC. The findings demonstrate that gut microbiota imbalance—characterized by abnormal enrichment of the *Megamonas* genus and an elevated B/B ratio—synergistically promotes EC progression by mediating lipid metabolism disorders (e.g., dysregulated glycerophospholipid metabolism, elevated serum triglyceride levels) and fostering a chronic inflammatory microenvironment. Notably, the strong correlation between microbial biomarkers (e.g., *Megamonas*) and host metabolic phenotypes (obesity severity, estrogen levels) highlights their potential as early diagnostic targets. For therapeutic interventions, probiotic supplementation may restore metabolic homeostasis and suppress inflammation by remodeling gut microbiota composition, while microbial metabolites such as SCFAs could indirectly reduce EC risk by enhancing intestinal barrier integrity and modulating immune microenvironments. Future research should integrate multi-omics data to explore precision strategies targeting the microbiota-metabolism axis (e.g., *Megamonas*-specific modulation or SCFA supplementation) and validate their long-term efficacy in reducing obesity-related EC incidence through multi-center clinical cohorts.

## 5 Limitations of the study

Although this study provides valuable insights into the impact of gut microbiota and lipid metabolism on the progression of EC in overweight individuals, there are still some shortcomings in both the interpretation of the results and the study design. First, the sample size of this study is relatively small, which somewhat limits the statistical power of the results. This limitation may hinder the study’s ability to fully demonstrate the true clinical effects when exploring the complex relationship between gut microbiota, lipid metabolism, and EC. Therefore, to improve the reliability and generalizability of the 12 findings, future research should consider increasing the sample size and adopting a multi-center study design to enhance the external validity of the results, minimizing potential biases related to regional differences or population characteristics. Additionally, while this study investigates the impact of gut microbiota and lipid metabolism on the progression of endometrial cancer, the analysis of the underlying mechanisms remains somewhat superficial. The article mentions that gut dysbiosis might contribute to tumor progression by affecting intestinal permeability, immune response, and other pathways. However, it does not provide a detailed explanation of how these mechanisms might influence the occurrence and development of EC at the molecular level through specific signaling pathways, such as MAPK, PI3K/Akt, and others. A deeper understanding of these mechanisms could help elucidate the causal relationship between gut microbiota, lipid metabolism, and EC. Moreover, the study did not fully address the potential limitations related to diet, which is a crucial factor in influencing gut microbiota composition and metabolism. The diet of participants was not adequately controlled, and this may have introduced variability in the results. As dietary habits can significantly affect gut microbiota diversity and metabolic pathways, it is important for future studies to consider the impact of diet more thoroughly. Integrating dietary influences into the research framework could help mitigate bias and provide a more accurate understanding of the relationship between gut microbiota, lipid metabolism, and EC. Future research could incorporate multi-dimensional data analysis, combining specific microbiota changes with host metabolism and immune responses, and taking diet into account, to explore the mechanisms in a more comprehensive and multi-layered manner. This approach would provide a more robust and detailed explanation of the mechanisms involved in EC progression.

## 6 Conclusion

This study reveals the critical interplay between gut microbiota dysbiosis (e.g., *Megamonas* enrichment) and lipid dysregulation in driving EC progression in overweight individuals. Specifically, the enrichment of *Megamonas*—a key microbial biomarker—exhibits strong correlations with tumor advancement and host metabolic disturbances. Mechanistically, *Megamonas* promotes lipid metabolic reprogramming (e.g., disrupted glycerophospholipid metabolism and elevated triglycerides) and fosters a chronic inflammatory microenvironment through TLR4/NF-κB signaling, thereby accelerating EC pathogenesis. Clinically, *Megamonas*-associated metabolites (e.g., palmitoleic acid) correlate with obesity severity and hyperestrogenemia, highlighting their dual diagnostic and therapeutic potential. Future research should prioritize multi-omics approaches to develop precision therapies targeting the microbiota-metabolism axis and validate their efficacy in large-scale clinical trials.

## Data Availability

The datasets for this study can be found in the Genome Sequence Archive at the National Genomics Data Center (Nucleic Acids Res 2024), China National Center for Bioinformation / Beijing Institute of Genomics, Chinese Academy of Sciences (GSA: PRJCA034591) [https://ngdc.cncb.ac.cn/gsa].
