# Correction: The impact of altered gut microbiota and lipid metabolism on the progression of endometrial cancer in overweight populations

**DOI:** 10.3389/fendo.2025.1681661

**Published:** 2025-08-29

**Authors:** Jiayu Chen, Haochen Peng, Yawen Shao, Zhenzhen Wu

**Affiliations:** ^1^ Gansu Province Maternal and Child Health Hospital (Gansu Central Hospital) Women’s Health Department, Lanzhou, China; ^2^ First School of Clinical Medicine, Gansu University of Chinese Medicine, Lanzhou, China

**Keywords:** overweight, endometrial cancer, gut microbiota, gut microbial ecosystem, lipid metabolism

Affiliation 1 was erroneously assigned to author “Jiayu Chen”. The correct affiliation number should be “2”. Affiliation 2 was erroneously assigned to author “Yawen Shao”. The correct affiliation number should be “1”.

The correct author affiliation list appears below.

“Jiayu Chen^2^, Haochen Peng^1^, Yawen Shao^1^, Zhenzhen Wu^1, 2*^.

^1^Gansu Province Maternal and Child Health Hospital (Gansu Central Hospital) Women’s Health Department, Lanzhou, China

^2^First School of Clinical Medicine, Gansu University of Chinese Medicine, Lanzhou, China”

The original version of this article has been updated.

