# Correction: The impact of altered gut microbiota and lipid metabolism on the progression of endometrial cancer in overweight populations

**DOI:** 10.3389/fendo.2025.1725835

**Published:** 2025-12-23

**Authors:** Jiayu Chen, Haochen Peng, Yawen Shao, Zhenzhen Wu

**Affiliations:** 1 Gansu Province Maternal and Child Health Hospital (Gansu Central Hospital) Women's Health Department, Lanzhou, China; 2 First School of Clinical Medicine, Gansu University of Chinese Medicine, Lanzhou, China

**Keywords:** overweight, endometrial cancer, gut microbiota, gut microbial ecosystem, lipid metabolism

In the published article, there was an error. The inclusion and exclusion criteria in this article were contradictory.

A correction has been made to **Materials and methods**, *Inclusion and exclusion criteria, and grouping*, 2. This sentence previously included these two points:

“(3). No use of antibiotics, probiotics, or other medications that may affect gastrointestinal function within 4 weeks prior to enrollment. (4). No history of diarrhea or other gastrointestinal diseases within 4 weeks prior to enrollment”

These two points have been removed.

The original article has been updated.

