# Supplementary material for: The impact of altered gut microbiota and lipid metabolism on the progression of endometrial cancer in overweight populations
Source: Front Endocrinol (Lausanne). 2025 Jul 31;16:1610534. doi: 10.3389/fendo.2025.1610534 (PMC12368975; doi:10.3389/fendo.2025.1610534)
Supplement: Supplementary file 1 [file Table1.docx]

| **Component** | **Volume** |
| --- | --- |
| 5xFastPfu Buffer | 4μl |
| 2.5 mM dNTPs | 2μl |
| Forward Primer 338F (5 μM) | 0.8μl |
| Reverse Primer 806R (5 μM) | 0.8μl |
| FastPfu Polymerase | 0.4μl |
| Template DNA | 30ng |

**Table S1: PCR Amplification System**

| Time (min) | Mobile Phase B Percentage (%) |
| --- | --- |
| 0.0 | 5 |
| 0.5 | 5 |
| 9.5 | 100 |
| 11.5 | 100 |
| 12.0 | 5 |
| 15.0 | 5 |

**Table S2:Gradient Elution Program**

The injection volume is 5 µl per sample.
