# Supplementary material for: The impact of altered gut microbiota and lipid metabolism on the progression of endometrial cancer in overweight populations
Source: Front Endocrinol (Lausanne). 2025 Jul 31;16:1610534. doi: 10.3389/fendo.2025.1610534 (PMC12368975; doi:10.3389/fendo.2025.1610534)
Supplement: Supplementary file 2 [file DataSheet1.doc]

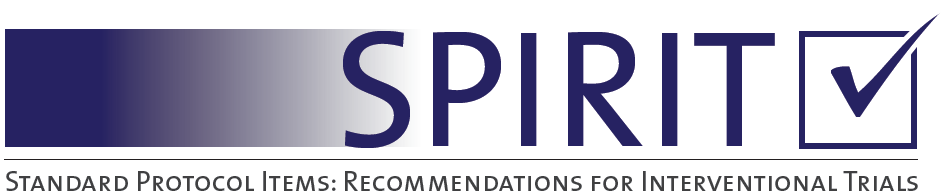


SPIRIT 2013 Checklist: Recommended items to address in a clinical trial protocol and related documents*

| Section/item | ItemNo | Description |
| --- | --- | --- |
| **Administrative information** | | |
| Title | 1 | Page 1 / Lines 1 - 3 |
| Trial registration | 2 | Page 2 / Lines 30 |
| Protocol version | 3 | Page 5 / Lines 32 |
| Funding | 4 | Page 7 / Lines 18 - 22 |
| Roles and responsibilities | 5a | Page 7 / Lines 25 - 28 |
|  | 5b | Page 1 / Lines 17 - 22 |
|  | 5c | N/A |
| Introduction |  |  |
| Background and rationale | 6a | Page 3 / Lines 3 - 39 |
|  | 6b | N/A |
| Objectives | 7 | Page 3 / Lines 35 - 39 |
| Trial design | 8 | Page 4 / Lines 13 - 35 |
| Methods: Participants, interventions, and outcomes | | |
| Study setting | 9 | Page 4 / Lines 14 |
| Eligibility criteria | 10 | Page 8 - 9 |
| Interventions | 11a | Page 4 / Lines 17 - 24 |
|  | 11b | Page 4 / Lines 37 - 42 |
|  | 11c | N/A |
|  | 11d | Page 4 / Lines 42 - 46 |
| Outcomes | 12 | Page 5 / Lines 4 - 15 |
| Participant timeline | 13 | see Figure 1 |
| Sample size | 14 | N/A |
| Recruitment | 15 | N/A |
| **Methods: Assignment of interventions (for controlled trials)** | | |
| Allocation: |  |  |
| Sequence generation | 16a | N/A |
| Allocation concealment mechanism | 16b | N/A |
| Implementation | 16c | N/A |
| Blinding (masking) | 17a | N/A |
|  | 17b | N/A |
| **Methods: Data collection, management, and analysis** | | |
| Data collection methods | 18a | N/A |
| Data management | 19 | N/A |
| Statistical methods | 20a | N/A |
|  | 20b | N/A |
|  | 20c | N/A |
| **Methods: Monitoring** | | |
| Data monitoring | 21a | N/A |
|  | 21b | N/A |
| Harms | 22 | N/A |
| Auditing | 23 | N/A |
| Ethics and dissemination | | |
| Research ethics approval | 24 | Page 7 / Lines 6 - 9 |
| Protocol amendments | 25 | N/A |
| Consent or assent | 26a | N/A |
|  | 26b | N/A |
| Confidentiality | 27 | N/A |
| Declaration of interests | 28 | Page 7 / Lines 18 |
| Access to data | 29 | N/A |
| Ancillary and post-trial care | 30 | N/A |
| Dissemination policy | 31a | N/A |
| Appendices |  |  |
| Informed consent materials | 32 | see Supporting Information file |
| Biological specimens | 33 | see Supporting Information file |

*It is strongly recommended that this checklist be read in conjunction with the SPIRIT 2013 Explanation & Elaboration for important clarification on the items. Amendments to the protocol should be tracked and dated. The SPIRIT checklist is copyrighted by the SPIRIT Group under the Creative Commons “[Attribution-NonCommercial-NoDerivs 3.0 Unported](http://www.creativecommons.org/licenses/by-nc-nd/3.0/)” license.
